# miR-541 Contributes to Microcystin-LR-Induced Reproductive Toxicity through Regulating the Expression of p15 in Mice

**DOI:** 10.3390/toxins8090260

**Published:** 2016-09-06

**Authors:** Xiannan Meng, Ling Zhang, Xiang Chen, Zou Xiang, Dongmei Li, Xiaodong Han

**Affiliations:** 1 Immunology and Reproduction Biology Laboratory & State Key Laboratory of Analytical Chemistry for Life Science, Medical School, Nanjing University, 22 Hankou Road, Nanjing 210093, Jiangsu, China; mengxian@yeah.net (X.M.); 15850581665@163.com (L.Z.); chenxiang910110@163.com (X.C.); 2 Jiangsu Key Laboratory of Molecular Medicine, Medical School, Nanjing University, 22 Hankou Road, Nanjing 210093, Jiangsu, China; 3 Department of Health Technology and Informatics, Faculty of Health and Social Sciences, The Hong Kong Polytechnic University, Hung Hom, Kowloon, Hong Kong, China; xiang.y.zou@polyu.edu.hk

**Keywords:** MC-LR, miR-541, p15, male reproduction, apoptosis

## Abstract

Microcystin-leucine arginine (MC-LR) is a harmful cyanotoxin produced by cyanobacteria. MC-LR can exert endocrine-disrupting activities in many organisms. We have previously demonstrated that MC-LR exerts both acute and chronic reproductive toxicity in male mice, resulting in a decline in sperm quality and damage to testicular structure. Moreover, we also observed extensive alterations in a panel of microRNAs in spermatogonial cells after exposure to MC-LR. In this study, we have confirmed that miR-541 was significantly increased both in GC-1 cells (in vitro) and in mouse testes (in vivo) after exposure to MC-LR. Our data support that p15 was the target gene of miR-541. Increase in miR-541 led to a reduction of p15 and murine double minute2 (MDM2), promoting the activation of p53 signaling and MC-LR-mediated cell apoptosis. Moreover, cells responded to MC-LR with reduced viability and increased apoptosis. Consistently, inhibiting miR-541 could upregulate the expression of p15 and MDM2, resulting in the downregulation of phospho-p53. Downregulation of miR-541 promoted cell viability by reducing MC-LR-induced cell apoptosis. In conclusion, we demonstrate here a crucial role for miR-541 in MC-LR-induced toxic effects on the reproductive system, in an attempt to provide a rational strategy for the diagnosis and treatment of MC-LR-induced impairment in the reproductive system.

## 1. Introduction

The presence of cyanobacteria in water bodies is common, and the development of cyanobacterial blooms has become more frequent and severe in the past decades [1,2,3,4,5]. Microcystins (MCs) are a family of cyclic heptapeptide compounds produced by cyanobacterial species [6,7] and have been the subject of worldwide concern in recent decades [8]. Owing to their accumulation in aquatic creatures, MCs also present health hazards to humans and animals higher up in the food chain [9].

So far, more than 80 variants of MCs have been identified in freshwater systems [10], among which microcystin-leucine arginine (MC-LR) is the most common MC species [11]. In addition, MC-LR has been detected at up to 0.39 μg/L in the serum of chronically exposed humans [12]. The World Health Organization (WHO) proposed 1 μg/L as an upper limit of the concentration of MC-LR (Microcystin-leucine arginine) in drinking water. It is reported that MC-LR can affect many organs including the thyroid [13], brain [14], liver [15], heart [16], intestine [17], kidneys [18], and reproductive organs [19].

We previously demonstrated that MC-LR showed acute and chronic toxicity in male reproduction, resulting in a decline in sperm quality and changes in the histological structure of testes [20]. We also found that MC-LR can regulate the expression of specific genes and induce apoptosis and proliferative responses in spermatogenic cells both in vivo [21] and in vitro [22]. However, the molecular mechanisms that may account for the reproductive toxicity of MC-LR remain to be clarified.

microRNA (miRNA) is small non-coding RNA 18 to 24 nucleotides in length. It can regulate gene expression and modify the translation process by binding to the 3′-UTR of mRNA [23]. To date, miRNA has been demonstrated to play key roles in diverse cellular processes, including differentiation, proliferation, and cycling. It is also implicated in various pathologies such as the development of cancer [24].

It is reported that miRNA plays an important role in MC-induced cancer development and embryogenesis [25,26]. We have previously found that a total of 101 miRNAs were substantially changed in GC-1 cells upon treatment with MC-LR [27]. Among these regulated miRNAs, miR-541 stood out as the most significantly upregulated. In the current work, we confirmed that miR-541 can directly target the 3′-UTR of p15, a cyclin-dependent kinase (CDK) inhibitor, which is proposed to be involved in cell cycle regulation [28]. p15 can block cell cycle progression and influence tumorigenesis, senescence, and ageing [29].

In conclusion, we revealed the roles of miR-541 in apoptotic regulation and its potential contribution to MC-LR induced toxicity in spermatogonial cells, which may provide a mechanistic explanation for reproductive malfunction including declines in sperm quality.

## 2. Results

### 2.1. The Expression of p15 Is Regulated by miR-541 in GC-1 Cells

**Figure 1:**
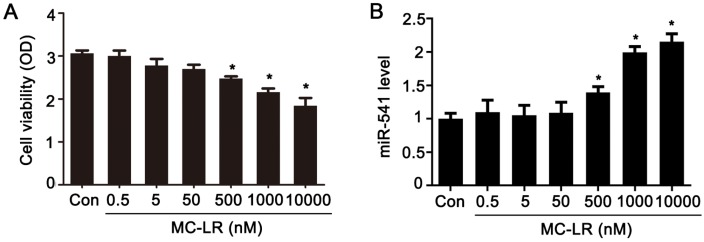

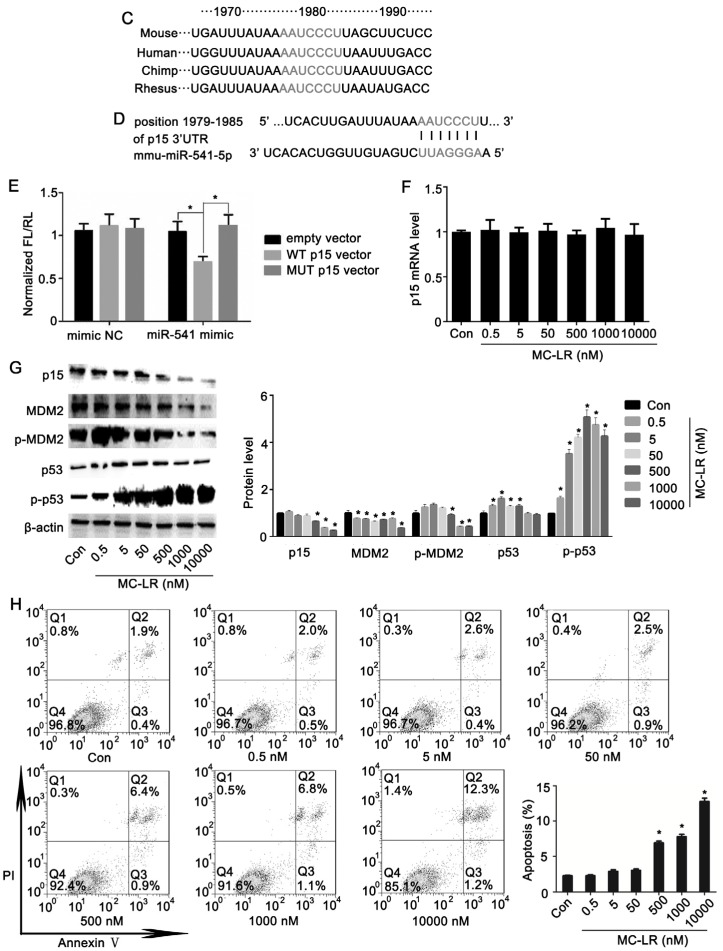
Effects of MC-LR (Microcystin-leucine arginine) on GC-1 cells. (**A**) Measurement of cell viability was carried out with the CCK-8 assay; (**B**) mRNA levels of miR-541 were measured with qRT-PCR in GC-1 cells after exposure to various concentrations of MC-LR for 24 h, as indicated; (**C**) alignment of orthologous segments of the 3′-UTR of p15, showing the conserved match to the miR-541 seed, and the seed matches (gray); (**D**) nucleotide sequences of murine miR-541 and the 3′-UTR of p15 region, which encompasses the miR-541 seed match region; (**E**) luciferase reporter assays were carried out in 293T cells following co-transfection of miR-541-mimic negative control (mimic-NC) or miR-541-mimic (mimic) together with wild-type (WT) p15 vector, mutant (MUT) p15 vector, or empty vector, as indicated. Firefly luciferase activity was normalized based on the Renilla luciferase activity; (**F**) The mRNA levels of p15 were measured with qRT-PCR in GC-1 cells exposed to various concentrations of MC-LR for 24 h, as indicated; (**G**) the protein levels of p15, murine double minute2 (MDM2), phospho-MDM2 (p-MDM2), p53, and phospho-p53 (p-p53) in GC-1 cells treated with various concentrations of MC-LR were measured by Western blot. The expression levels were quantified with ImageJ (right panels). β-actin was used as a loading control (*n* = 3); (**H**) GC-1 cells were treated with various concentrations of MC-LR for 24 h, as indicated. Cells were stained with FITC-coupled Annexin V and propidium iodide (PI). Both early and late apoptotic cells were revealed by flow cytometric dot plots (Q2 + Q3) and the data were summarized (right panels; *n* = 3). Results are expressed as means ± S.D. * *p* < 0.05.

We treated GC-1 cells with various concentrations of MC-LR for 24 h. Cell viability was significantly decreased at an MC-LR concentration of 500 nM or above (Figure 1A). The expression of miR-541 was significantly upregulated after exposure to MC-LR at these concentrations (Figure 1B).

Next, we tried to predict the potential targets of miR-541 from TargetScan Human V 6.2, miRanda, and miRBase. We searched potential targeting sites of miRNAs within the 3′-UTRs of spermatogenesis-associated mRNAs. p15 stood out as one of the target mRNAs of miR-541. The binding site for miR-541 in the 3′-UTR of p15 was highly conserved (Figure 1C,D).

We next tried to confirm the binding of miR-541 to the 3′-UTR of p15 in 293T cells using the dual luciferase reporter gene assay system. p15 3′-UTR and p15 3′-UTR with mutations in the predicted miR-541 binding site were cloned separately into the luciferase reporter system (GV306 vector). The GV306 vectors were then co-transfected with either miR-541 mimic or its negative control. Luciferase activity of the system containing wild-type, but not mutated, p15 3′-UTR was substantially decreased after co-transfection with miR-541 mimic (Figure 1E), which supported the prediction that p15 was the target gene of miR-541.

The mRNA levels of p15 were not changed in GC-1 cells upon treatment with MC-LR (Figure 1F). In contrast, at the protein level MC-LR profoundly suppressed p15 expression (Figure 1G). Our results suggest that miR-541 may regulate the expression of p15 in a post-transcriptional manner.

### 2.2. p15 May Regulate Apoptosis via the MDM2-p53 Pathway in GC-1 Cells

A recent study showed that the apoptosis of smooth muscle cells was increased in p15 knockout mice. The authors proposed that p15 may regulate apoptosis by targeting an MDM2-stabilizing kinase to enhance p53 ubiquitination and degradation [30]. This prompted us to explore whether the levels of MDM2 (murine double minute2) and p53 were affected in the p15-deficient spermatogonial cells. We found that the expression of total MDM2, phospho-MDM2 and p15 were consistently decreased in MC-LR-treated GC-1 cells (Figure 1G). On the contrary, the expression of phospho-p53 increased at identical settings. Furthermore, 500 nM MC-LR was able to induce apoptosis of GC-1 cells (Figure 1H).

### 2.3. Inhibition of miR-541 Protects GC-1 Cells from MC-LR-Induced Cell Death in Vitro

**Figure 2:**
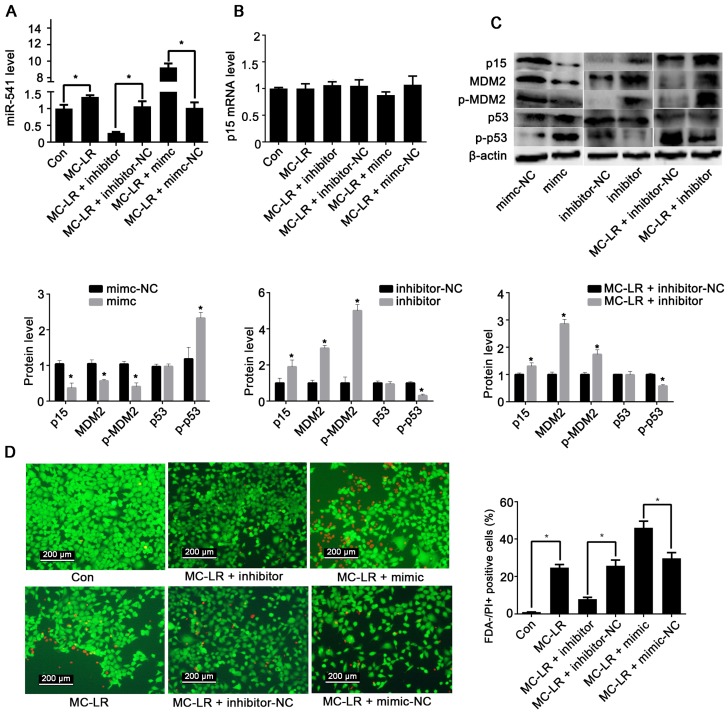

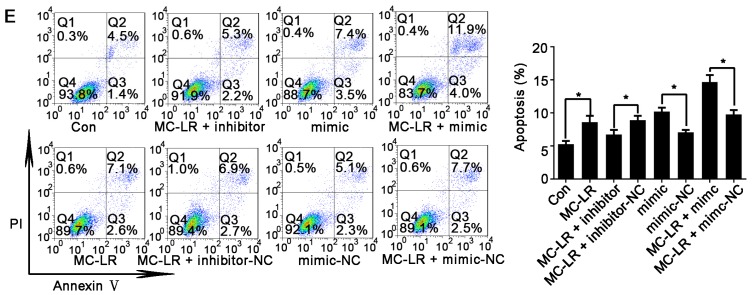
The effects of miR-541 on GC-1 cells. (**A**,**B**) Six hours after GC-1 cells were transfected with miR-541-inhibitor (inhibitor), miR-541-inhibitor negative control (inhibitor-NC), miR-541-mimic (mimic), or miR-541-mimic negative control (mimic-NC), cells were incubated with 500 nM MC-LR for 24 h, followed by measurement of the mRNA levels of miR-541 (**A**) and p15 (**B**) with qRT-PCR; (**C**) Six hours after GC-1 cells were transfected with mimic-NC, mimic, inhibitor-NC, or inhibitor, cells were incubated with or without 500 nM MC-LR for 24 h. The protein levels of p15, murine double minute2 (MDM2), phospho-MDM2 (p-MDM2), p53, and phospho-p53 (p-p53) were measured by Western blot. Protein expression levels were quantified with ImageJ (lower panels; *n* = 3). β-actin was used as a loading control; (**D**) GC-1 cells were transfected with mimic, mimic-NC, inhibitor, or inhibitor-NC, followed by treatment with 500 nM MC-LR for 24 h. The viability of GC-1 cells was revealed by FDA-PI staining. Uptake of FDA (green) indicates live cells, while uptake of propidium iodide (PI; red) indicates late apoptotic and dead cells. The percentages of FDA-/PI+ positive cells were quantified (right panels; *n* = 3); (**E**) GC-1 cells were stained with FITC-coupled Annexin V and propidium iodide (PI). Both early and late apoptotic cells (Q2 + Q3) were examined with flow cytometry. The percentage of apoptotic cells was calculated and summarized (lower panels; *n* = 3). Results are expressed as means ± S.D. * *p* < 0.05.

To examine the regulatory effects of miR-541 and p15 on cellular functions, we transfected GC-1 cells with miR-541-inhibitor, miR-541-mimic, and their negative controls followed by exposure to 500 nM MC-LR for 24 h. miR-541 was decreased in cells transfected with miR-541-inhibitor and highly increased in cells transfected with miR-541-mimic (Figure 2A). The expression of p15 mRNA remained unchanged following transfection with either miR-541-mimic or the inhibitor (Figure 2B). We next measured the effect of modulation of miR-541 on the protein levels of p15 and a group of associated regulatory proteins. Upregulating miR-541 suppressed the expression of p15, MDM2 (murine double minute2) and phospho-MDM2, while it increased the level of phospho-p53 (Figure 2C). Exactly the opposite trends were observed if intracellular miR-541 was suppressed. Furthermore, forced expression of miR-541 promoted MC-LR-induced cell apoptosis, whereas inhibiting miR-541 suppressed apoptosis (Figure 2D,E).

### 2.4. MC-LR Regulates the Expression of miR-541 and p15 in Vivo

**Figure 3:**
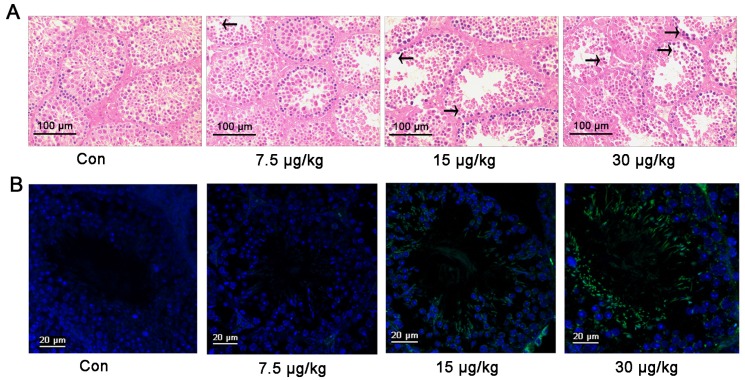

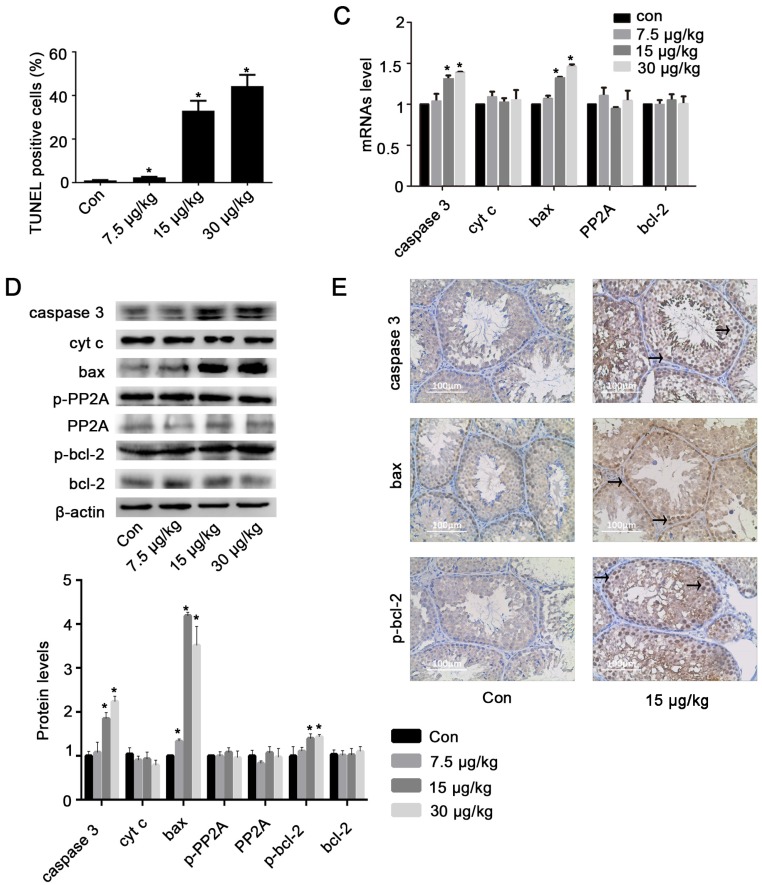
Toxic effects of MC-LR on testicular tissue. Mice were intraperitoneally injected with various concentrations of MC-LR. (**A**) The impact of MC-LR on testicular tissue structural changes was determined by H&E staining. The arrows indicate the loss of spermatogenic cells; (**B**) Fluorescent images of testicular tissues following double staining for transferase-mediated deoxyuridine triphosphate-biotin nick end labeling (TUNEL) (yellow) and the nuclear stain 4′, 6-diamidino-2-phenylindole (DAPI) (blue) are shown. The percentages of TUNEL positive cells were quantified (lower panels; *n* = 3); (**C**) The mRNA levels of caspase 3, cytochrome c (cyt c), bcl-2-associated x (bax), protein phosphatase 2A (PP2A), and B-cell lymphoma-2 (bcl-2) were measured with qRT-PCR in testicular tissues exposed to various concentrations of MC-LR, as indicated; (**D**) The protein levels of caspase 3, cyt c, bax, PP2A, phospho-PP2A (p-PP2A), bcl-2, and phospho-bcl-2 (p-bcl-2) in testicular tissue treated with various concentrations of MC-LR were measured by Western blot. The expression levels were quantified with ImageJ (lower panels; *n* = 3). β-actin was used as a loading control; (**E**) Caspase 3, bax, and p-bcl-2 in mouse testicular tissue were revealed by immunohistochemistry. Positive cells are marked by an arrow. Results are expressed as means ± S.D. * *p* < 0.05.

Two weeks after exposure to MC-LR, mouse testicular tissue structure was disrupted, as evidenced by H&E staining. The testicular structure of mice that received 7.5 μg/kg MC-LR altered modestly. In contrast, the spermatogenic epithelium became loosened in its organization and disordered spermatogenic cell arrangement was pronounced upon treatment with 15 μg/kg and 30 μg/kg MC-LR (Figure 3A).

The number of apoptotic testicular cells increased with the increase of MC-LR concentration (Figure 3B). We next analyzed the mRNA and protein expression of some apoptosis-associated genes in mouse testes following MC-LR treatment. The mRNA expression of caspase3 and bax was upregulated at 15 μg/kg and 30 μg/kg MC-LR (Figure 3C). The protein levels of caspase3, bax and phospho-bcl-2 were significantly increased (Figure 3D). Furthermore, the expression of caspase3, bax, and phospho-bcl-2 was significantly increased in the spermatogonial cells from mice that received 15 μg/kg MC-LR (Figure 3E). These results suggested that cell apoptosis induction by MC-LR may be regulated by altered expression of apoptosis-associated genes in vivo.

**Figure 4:**
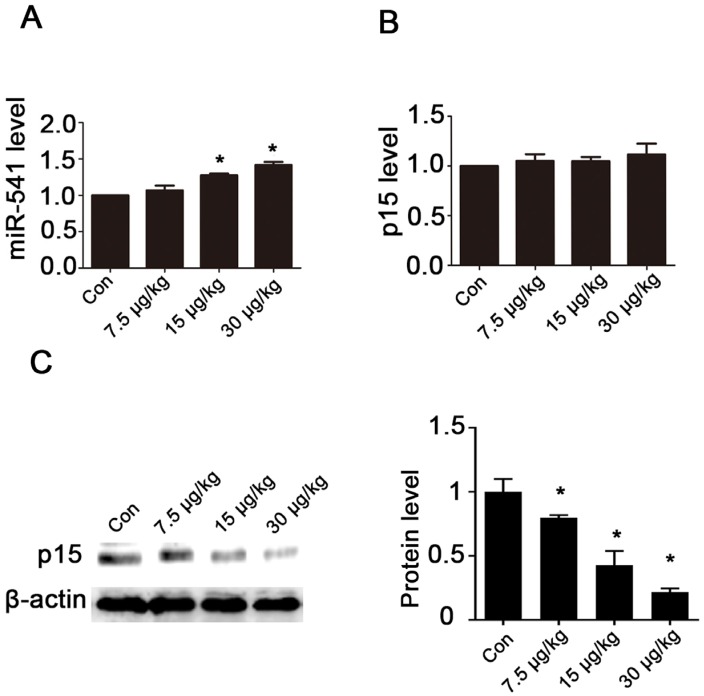
The effects of MC-LR on the expression of miR-541 and p15 in mouse testes. (**A**,**B**) Mice were intraperitoneally injected with various concentrations of MC-LR. The mRNA levels of miR-541 (**A**) and p15 (**B**) in testes were measured with qRT-PCR; (**C**) The protein levels of p15 in testicular tissues were measured by Western blot. The expression of p15 was quantified with ImageJ (right panels; *n* = 3). β-actin was used as a loading control. Results are expressed as means ± S.D. * *p* < 0.05.

The expression of miR-541 and p15 was determined in testicular tissue from either control mice or mice treated with MC-LR. miR-541 was significantly upregulated when higher concentrations of MC-LR (15 μg/kg and 30 μg/kg) were delivered (Figure 4A). The mRNA levels of p15 remained unchanged (Figure 4B) and the protein levels of p15 were significantly downregulated (Figure 4C). These results were consistent with our in vitro findings.

### 2.5. Inhibition of miR-541 Protects GC-1 Cells from MC-LR-Induced Cell Death in Vivo

**Figure 5:**
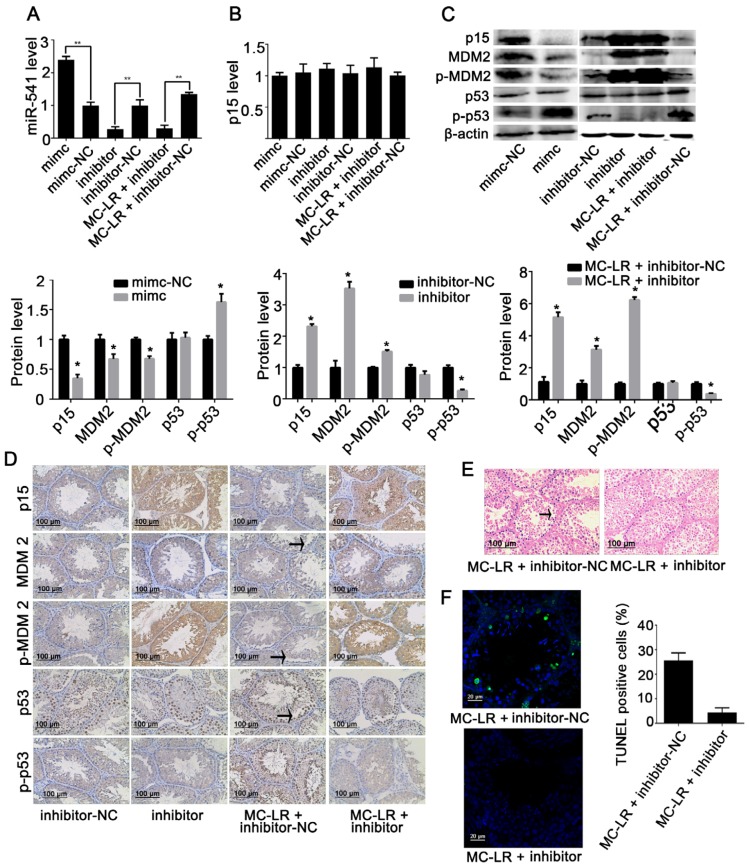
Validation of the functions of miR-541 in mouse testes. Mice received miR-541-inhibitor (inhibitor), miR-541-mimic (mimic), miR-541-inhibitor negative control (inhibitor-NC), or miR-541-mimic negative control (mimic-NC) via efferent duct injection, with or without intraperitoneal administration of 15 μg/kg body weight MC-LR. The mRNA levels of miR-541 (**A**) and p15 (**B**) were measured with qRT-PCR; (**C**) The protein levels of p15, murine double minute2 (MDM2), phospho-MDM2 (p-MDM2), p53, and phospho-p53 (p-p53) were measured by Western blot. The expression levels were quantified with ImageJ (lower panels; *n* = 3). β-actin was used as a loading control; (**D**) The expression of p15, MDM2, p-MDM2, p53, and p-p53 in testes was observed by immunohistochemistry. Positive cells are marked by an arrow; (**E**) The testicular tissue structure was determined by H&E staining. The arrow indicates the loss of spermatogenic cells; (**F**) Fluorescent images of testicular tissues following double staining for TUNEL (yellow) and the nuclear stain DAPI (blue) are shown. The percentages of TUNEL positive cells were quantified (right panels; *n* = 3). Results are expressed as means ± S.D. * *p* < 0.05.

Constructs containing miR-541-mimic, miR-541-inhibitor, and their negative controls were delivered through the efferent ducts. We found that the mimic increased the expression of miR-541, whereas the inhibitor suppressed the expression of miR-541. Interestingly, miR-541-inhibitor was potent in antagonizing MC-LR-mediated upregulation of miR-541 (Figure 5A). However, mRNA expression of p15 was not modulated by the regulation of miR-541, which was consistent with our in vitro data (Figure 5B). We further investigated the effect of manipulated miR-541 expression on the protein levels of p15, MDM2, phospho-MDM2, p53 and phospho-p53 either with or without exposure to MC-LR by Western blot. Upregulating the expression of miR-541 suppressed the levels of p15, MDM2, and phospho-MDM2 while it increased the level of phospho-p53 (Figure 5C). Suppression of miR-541 was correlated with upregulation of p15, MDM2 and phospho-MDM2, but downregulation of phospho-p53 in the testicular tissue irrespective of prior exposure of the mice to MC-LR (Figure 5C). These results were also supported by immunohistochemical staining (Figure 5D). Furthermore, inhibiting miR-541 effectively restored testicular structures, mature sperm counts, and testicular cell viability following exposure to MC-LR (Figure 5E,F).

## 3. Discussion

MC-LR is a potent reproductive system toxin present in water bodies [22] and can mimic the effects of endocrine disrupting chemicals [31,32]. Our previous work has confirmed that low-dose, chronic exposure to MC-LR decreases the levels of serum testosterone and increases the levels of luteinizing hormone (LH) and follicle-stimulating hormone (FSH) [20]. Testosterone is a critical germ cell survival factor that regulates the apoptotic mechanisms of testicular germ cells both in vivo and in vitro [33,34]. Taken together, it is suggested that MC-LR may induce reproductive toxicity through decreasing the expression of testosterone as an endocrine disrupting chemical. It is reported that MC-LR at as low as 50 nM can produce sufficient toxicity, resulting in viability loss in rat primary spermatogonia [22]. Moreover, it is found that the proliferative responses of GC-1 cells were significantly reduced after exposure to MC-LR at concentrations from 500 nM to 100 μM, whereas lower concentrations did not achieve the observed effects [27]. The changes in cell morphology were more pronounced when the concentrations of MC-LR were above 500 nM [27]. Therefore, we finally chose 500 nM to investigate the effect of MC-LR in vivo. In the current study, there were no significant effects observed in GC-1 cells with MC-LR concentrations up to 50 nM (Figure S1). The effects became substantial when MC-LR concentrations of at least 500 nM were employed (Figure 1A). These observations highlight different levels of sensitivity between GC-1 cells and spermatogonia in response to MC-LR. The current study has demonstrated that MC-LR can be taken up by GC-1 cells (Figure S2), although up to now no evidence supports the expression of organic anion-transporting polypeptides (Oatps) on GC-1 cells. It is documented that MC-LR can migrate into rat spermatogonia, which express Oatps (Oatp1a5, -3a1, -6b1, -6c1, and -6d1; the expression of these Oatps, especially Oatp3a1, is modulated by MC-LR [22]. Moreover, it is reported that long-term drinking of water containing low dose MC-LR promotes follicle atresia and eventually leads to female subfertility [35]. In this study, we aimed to establish an acute toxicity model of male mice by intraperitoneal injection of MC-LR in order to explore the mechanisms of MC-LR-induced testicular cell apoptosis. We believe that this route would efficiently deliver MC-LR to testicular tissues. In this study, we proposed that miRNA may play an important role in MC-LR-induced pathology. miRNA, which regulates gene expression post-transcriptionally, plays a crucial role in spermatogenesis [36]. Indeed, our previous study has revealed altered expression of miRNAs in GC-1 cells after exposure to MC-LR [27]. Therefore, we were intrigued to identify specific miRNA that could regulate MC-LR-induced toxicity.

In this study, we confirmed that miR-541 was upregulated after treatment of the cells with MC-LR. miR-541 was significantly increased in GC-1 cells after exposure to 500 nM MC-LR. The treatment of MC-LR resulted in cell apoptosis and a destruction of testicular structure. MC-LR has been demonstrated to be able to induce the expression of apoptosis-related proteins such as p53, bax, and caspase3 [37,38,39]. We also confirmed these findings both in vivo and in vitro.

Effective inhibition of mRNA translation by miRNAs is known to be mediated by the specific site at 3′-UTR [40]. The 3′-UTR of p15 has a miR-541 binding sequence. p15, a member of the INK4 family of cyclin-dependent kinase inhibitors, mediates signals essential for maintaining cell cycle [41] and cell apoptosis [30]. Moreover, p15 plays an important role in spermatogenesis [42]. In our study, we demonstrated that miR-541 could mediate post-transcriptional regulation of p15 in testes. The specific function of miR-541 and p15 in germline aroused our interest. It is reported that apoptotic p15-deficient human coronary artery smooth muscle cells (HCASMC) significantly reduced expression of both total MDM2 and phosphorylated MDM2 than apoptotic control cells [30]. MDM2 is a central regulator of the p53 pathway, which can inhibit the transcriptional activity, nuclear localization and protein stability of p53 [43]. The tumor suppressor p53 can be activated by cellular stresses such as DNA damage, oncogene activation or hypoxia, resulting in the initiation of a series of transcriptional programs that induce DNA repair, cell cycle arrest, apoptosis, or autophagy [44,45]. Phosphorylation of p53 is a key mechanism responsible for the activation of its functions in response to various stresses, and it is well documented that MDM2 plays an important role in regulating phosphorylation of p53 [46].

In our study, we found that miR-541 could induce cell apoptosis through diminishing the expression of p15, which would in turn downregulate both total MDM2 and phospho-MDM2, while increase phospho-p53. On the contrary, suppressing the expression of miR-541 could substantially attenuate MC-LR-induced cell apoptosis through increasing the expression of p15. Moreover, testicular structural damage caused by MC-LR was relieved by suppressing intracellular expression of miR-541.

In summary, the current study has revealed an important link among MC-LR, miR-541, and p15 in the male reproductive system using mouse models. We found that the MC-LR-induced toxic effects may be mediated by overexpression of miR-541, which promotes cell apoptosis by targeting p15 and a group of downstream apoptosis-associated proteins. Interfering with the intracellular expression of miR-541 may protect germ cells from the cytotoxic effects of MC-LR. These observations may suggest new biomarkers for the prevention and treatment of MC-induced male infertility.

## 4. Experimental Section

### 4.1. Chemicals and Reagents

Dulbecco’s modified Eagle’s medium (DMEM), penicillin and streptomycin sulfate were purchased from Sigma-Aldrich (St. Louis, MO, USA). The fetal bovine serum (FBS) was purchased from Dojindo Molecular Technologies (Kumamoto, Japan). SYBR GreenER qRT-PCR kit, Taqman microRNA assay and universal PCR Mastermix were purchased from Invitrogen (Shanghai, China). Rabbit anti-p15, rabbit anti-phospho-MDM2, rabbit anti-MDM2, mouse anti-p53, mouse anti-phospho-p53, mouse anti-MC-LR, rabbit anti-caspase3, mouse anti-bax, rabbit anti-phospho-bcl-2, and mouse anti-β-actin were purchased from Abcam (Cambridge, MA, USA). Rabbit anti-cytc c, rabbit anti-PP2A, mouse anti-phospho-PP2A, and rabbit anti-bcl-2 were purchased from Santa Cruz (LA, CA). Horseradish peroxidase-conjugated goat anti-rabbit/mouse IgG, which was used as the secondary antibody, were purchased from Boster (Wuhan, China). Enhanced chemiluminescence detection kit was purchased from Alpha (San Jose, CA, USA). Dual-Luciferase Reporter Assay System was purchased from Promega (Madison, WI, USA). Fluorescein diacetate (FDA) and propidium iodide (PI) were purchased from Sigma-Aldrich. The Annexin V–PI apoptosis assay kit was purchased from Nanjing KeyGen Biotech (Nanjing, China). MC-LR with a purity of ≥95% was purchased from Alexis Biochemicals (Lausen, Switzerland). The MC-LR was dissolved in methanol and diluted to 1 mg/mL or 10 mg/mL using phosphate-buffered saline (PBS) as a stock solution kept at −20 °C within six months.

### 4.2. Animals and Treatment

Six-week-old male BALB/c mice (specific pathogen-free, SPF) (*n* = 50) (18–22 g) were purchased from the Experimental Animal Center of the Academy of Military Medical Science, Beijing, China. The animal experiments were performed according to the Guide for the Care and Use of Laboratory Animals (The Ministry of Science and Technology of China, Nanjing, China, 2006) and all experimental protocols were approved under the animal protocol number SYXK (Su) 2009-0017 by the Animal Care and Use Committee of Nanjing University. Mice were individually housed in laminar flow cabinets under a specific pathogen-free environment with access to food and water libitum. The animals were acclimatized for one week before use, and maintained throughout at standard conditions: 50% relative humidity, 24 °C ± 1 °C, and 12:12 h light-dark circle.

Mice were randomized into 10 groups (*n* = 5 for each) (Table S1). Four groups of mice were each intraperitoneally injected with MC-LR at 7.5 (1/8 LD50), 15 (1/4 LD50) or 30 (1/2 LD50) μg/kg body weight, or an identical volume of 0.9% saline every day for 2 weeks. The MC-LR (stock solution) was dissolved in 400 μL saline adequately to achieve various concentrations desired. These four groups of mice were nine weeks old at the end of the treatment. The lentiviral vector is an efficient gene transduction system for mouse testes. We constructed lentiviral vectors that contain miR-541-mimic or miR-541-inhibitor, or their respective negative controls (inhibitor-NC and mimic-NC) separately (Genechem, Shanghai, China). The lentiviral vector was injected into mouse seminiferous tubules via the efferent ducts [47]. The seminiferous tubules of the testes of a mouse have a total injectable internal volume of about 10 μL (8 μL lentiviral vector solution and 2 μL trepan blue solution per testes) [48]. The concentration of the lentiviral vector was 1 × 10^8^ Transducing Units (TU)/mL (Figure S3). Another 4 groups of mice received lentiviral vectors containing mimic, inhibitor, mimic-NC, or inhibitor-NC separately. One week later, these four groups of mice received intraperitoneally 400 μL 0.9% saline solution every day for two weeks. The remaining two groups of mice were injected with inhibitor or inhibitor negative control. One week later, these two groups of mice were intraperitoneally injected with 15 μg/kg body weight MC-LR per day for two weeks (Table S2).

### 4.3. Cell Culture and Treatment

Mouse GC-1 cells, a well-established mouse cell line that shares characteristics of a stage between type B spermatogonia and primary spermatocytes, were obtained from Prof. Zuoming Zhou at Nanjing Medical University (Jiangsu, China). GC-1 cells were grown at 37 °C in a humidified 5% CO_2_ atmosphere in DMEM containing 10% FBS. Cells were subcultured every 2–3 days. MC-LR (stock solution) was dissolved in DMEM and applied to GC-1 cells at concentrations of 0, 0.5, 5, 50, 500, 1000, or 10,000 nM. For preparing lentiviral vector infected GC-1 cells, approximately 2 × 10^5^ cells were plated into each well of six-well culture plates and incubated for 6 h in DMEM supplemented with 10% FBS at 37 °C and 5% CO_2_. Next, 2 μL of the lentiviral vector solution was added to each well and the cells were incubated at 37 °C for 24 h. The concentration of the lentiviral vector solution was 1 × 10^8^ Transducing Units (TU)/mL. Cells were next incubated either in the absence or presence of 500 nM MC-LR for 24 h.

### 4.4. Cell Viability Assay

Cell viability was evaluated by the CCK-8 test according to a previous study [27]. CCK-8 contains WST-8, which can be deoxidized to a hydrosoluble formazan dye by mitochondrial dehydrogenase in living cells. Purified cells were digested and plated in a 96-well culture plate at 1 × 10^4^ cells per well and cultured in the serum-free growth medium. The cells were divided into eight groups followed by treatment with MC-LR at various concentrations (0.5, 5, 50, 500, 1000, or 10,000 nM) for 24 h. Following MC-LR treatment, 10 μL of CCK-8 solution was added to each well and the cells were incubated at 37 °C for a further 2 h. Absorbance, expressed as optical density (OD), was measured at 450 nm with a multidetection micro plate reader (Versamax, Chester, PA, USA).

### 4.5. Quantitative RT-PCR

One microgram of total RNA was used for cDNA synthesis. Reverse transcription was performed using the Superscript III first-strand synthesis system (Invitrogen, Carlsbad, CA, USA) or the Taqman microRNA everse transcription kit (Invitrogen) on a Veriti 96-Well Fast Thermal Cycler (Applied Biosystems, Grand Island, NY, USA). Quantitative PCR was performed using the SYBR GreenER qRT-PCR kit or the Taqman microRNA assay and universal PCR Mastermix using a 7900 HT Fast Real-Time PCR System (Applied Biosystems). All the procedures were repeated for three times. The relative quantification values for each miRNA and mRNA were calculated by the 2^−ΔΔ*C*t^ method using U6 and GAPDH as an internal reference, respectively. The sequences of mRNA primer pairs used in this assay were in the Supporting Information (Table S3).

### 4.6. Western Blot

Total protein was isolated from the cells following various treatments. Briefly, cells were washed three times with PBS and lysed in an ice-cold extraction buffer (50 mM Tris-HCl pH 7.4, 150 mM NaCl, 1% NP, 0.1% SDS, and 1× protease inhibitor cocktail). Protein concentration was determined by the Bradford method. Proteins were separated using sodium dodecyl sulfate-polyacrylamide gel electrophoresis (SDS-PAGE) and electroblotted onto a polyvinylidene fluoride (PVDF) membrane (Roche, Basel, Switzerland) by standard procedures. Transferred blots were incubated sequentially with antibodies including primary rabbit anti-p15, rabbit anti-phospho-MDM2, rabbit anti-MDM2, mouse anti-p53, mouse anti-phospho-p53, mouse anti-MC-LR, rabbit anti-caspase3, mouse anti-bax, rabbit anti-phospho-bcl-2, rabbit anti-cyt c, rabbit anti-PP2A, mouse anti-phospho-PP2A, rabbit anti-bcl-2, mouse anti-β-actin, and HRP-conjugated secondary antibodies. Protein bands were visualized with an enhanced chemiluminescence detection kit and recorded on a radiographic film (Alpha Innotech, San Jose, CA, USA).

### 4.7. FDA/PI Staining

The integrity of cell membranes was determined using FDA/PI staining according to a previous report [27]. Cells were placed on 96-well culture plates at 1 × 10^4^ cells/mL per well followed by exposure to various treatment conditions. Cell morphology was examined by staining with 5 μg/mL PI and 4 μg/mL FDA. All experiments consisted of three biological replicates, each examined at least in triplicate.

### 4.8. TUNEL Staining

TUNEL was performed as described previously [21]. The paraffin embedded testicular tissues were sectioned at 5 μm and placed on the glass slides at 37 °C overnight, followed by storage at 4 °C until standard processing. Briefly, 5 μm sections of testicular tissues were stained by H&E followed by deparaffinization, rehydration, fixation in 4% paraformaldehyde in PBS, permeabilization with Proteinase K, pre-equilibration, and labeling of DNA strand breaks with the reaction mixture for 1 h. Samples were visualized with DAPI nuclear stain in the mounting medium using a confocal microscope (Fluoview FV10i, Tyoko, Japan) to detect the localized apoptotic cells showing green fluorescence. Positive TUNEL POD labeling was prepared using the DNase treatment. After pretreatment, histological sections were incubated with DNase at 37 °C for 10 min to induce DNA strand breaks. Negative control was carried out by omitting terminal transferase from the labeling procedure.

### 4.9. Flow Cytometry Analysis.

The cells were washed twice with cold PBS and then suspended in 1 × Binding Buffer at a concentration of 1 × 10^6^ cells/mL. A total of 100 μL of the suspension (1 × 10^5^ cells) were transferred to a 5 mL tube and mixed with 5 μL of FITC-conjugated Annexin V and 5 μL PI. The mixture was gently vortexed and incubated for 15 min at room temperature (RT) in the dark. Next, 400 μL of 1 × Binding Buffer was added to each tube. The cells were analyzed by a BD FAC Scan flow cytometer (BD Bioscience, San Jose, CA, USA) using a 488 nm-laser and 530/30 nm and 585/42 nm bandpass filters for Fluorescein isothiocyanate isomer I (FITC) and PI, respectively.

### 4.10. Dual-luciferase Reporter Assay

Luciferase assays were carried out to confirm the interaction between miR-541 and the 3′-UTR of p15 (Table S4) using the GV306 plasmid vector (Figure S4). 293T cells were seeded into 24-well plates, co-transfected with 50 nM miR-541 or a scrambled mimic and 600 ng of a dual luciferase vector expressing the wild-type or mutant 3′-UTR p15 sequences. After 24 h, the luciferase activities were measured using the Dual- Luciferase Reporter Assay System (Promega, Madison, WI, USA) according to the manufacturer’s protocol. The ratio of firefly to Renilla luciferase signals was used to normalize firefly activity for intra-experimental transfection efficiency.

### 4.11. Histopathological Evaluation

The testes were fixed overnight at 4 °C in 4% (*w*/*v*) paraformaldehyde in PBS (pH = 7.4). They were dehydrated successively using 30%, 50%, 75%, 80%, 90%, 95%, and 100% ethanol. Next, the tissues were embedded in paraffin, sectioned at 5 μm, and stained with hematoxylin and eosin (H&E) for light microscopic examination.

### 4.12. Immunohistochemistry

The testes of mice were fixed, embedded, and cut into sections as described previously [49,50]. Subsequently, sections were deparaffinized with xylene (2 × 10 min), rehydrated with descending ethanol solutions (2 × 100%, 80%, 70%; each 5 min), and finally rinsed in distilled water for 5 min at RT. Thereafter, epitope retrieval was performed by incubating tissue sections for 10 min at 95 °C in 10 mM citric acid (pH 6.0), followed by 10 min washes with PBS. For quenching endogenous peroxidase activity, sections were first treated with 3% H_2_O_2_ for 10 min at RT. After washing the sections three times with PBS (5 min), unspecific binding sites were blocked with 5% normal calf serum (NCS) in PBS for 20 min at RT. The following incubation with primary antibodies diluted in 5% NCS/PBS was carried out overnight at 4 °C in a humidified chamber. Next, sections were washed three times with PBS (10 min) before the application of a biotinylated secondary antibody (1:500 dilution in 5% NCS/PBS). After a 2 h incubation period at RT, excess antibody was removed by three washes with PBS (10 min). For visualization of immunoreactivity, the DAB substrate (0.01% H_2_O_2_ and 0.05% DAB in H_2_O) was applied. To obtain optimal labeling intensity, staining development was followed under visual control and reaction was stopped by incubation in distilled water for 5 min. For nuclear counterstaining, sections were incubated in the hematoxylin solution (1 min) and rinsed in running tap water (10 min) for blue annealing. Subsequently, sections were dehydrated with ascending ethanol solutions (70% ethanol, 80% ethanol, 2 × 100% ethanol; 5 min), and finally cleared in xylene. Control sections were treated using the same incubation conditions, except that slides were incubated with 5% NCS/PBS instead of the primary antibody. Samples were subsequently coated with Entellan mounting medium, and examined using an Olympus CKX41 microscope equipped with bright-field light optics, an Olympus SC20 camera and CellA imaging software (Olympus, Hamburg, Germany).

### 4.13. Statistical Analysis.

All calculations and statistical analyses were performed using SPSS for Windows version 13.0 (SPSS, Chicago, IL, USA). One-way analysis of variance (ANOVA) was used to analyze the difference between groups, followed by the Dunnett’s *t* test. *p* < 0.05 is regarded as statistically significant.

## Supplementary Materials

The following are available online at www.mdpi.com/2072-6651/8/9/260/s1. Figure S1: Toxic effects of MC-LR on GC-1 cells; Figure S2: Intracellular MC-LR detected in GC-1 cells exposed to MC-LR for 24 h; Figure S3: Efferent duct injection; Figure S4: GV306 plasmid vector map; Table S1: Animals and treatment; Table S2: The sequence design of miR-541-inhibitor, inhibitor negtive control, miR-541-mimic and mimic negative control; Table S3: Oligonucleotide Sequences used in this study; Table S4: The wild type and mutant type of p15 3′UTR sequences containing the miR-541 binding site.
