# Supplementary material for: miR-541 Contributes to Microcystin-LR-Induced Reproductive Toxicity through Regulating the Expression of p15 in Mice
Source: Toxins (Basel). 2016 Sep 6;8(9):260. doi: 10.3390/toxins8090260 (PMC5037486; doi:10.3390/toxins8090260)
Supplement: Supplementary file 1 [file toxins-08-00260-s001.pdf]

Supplementary Materials: miR-541 Contributes to Microcystin-LR-Induced Reproductive Toxicity through Regulating the Expression of p15 in Mice

Xiannan Meng, Ling Zhang, Xiang Chen, Zou Xiang, Dongmei Li and Xiaodong Han

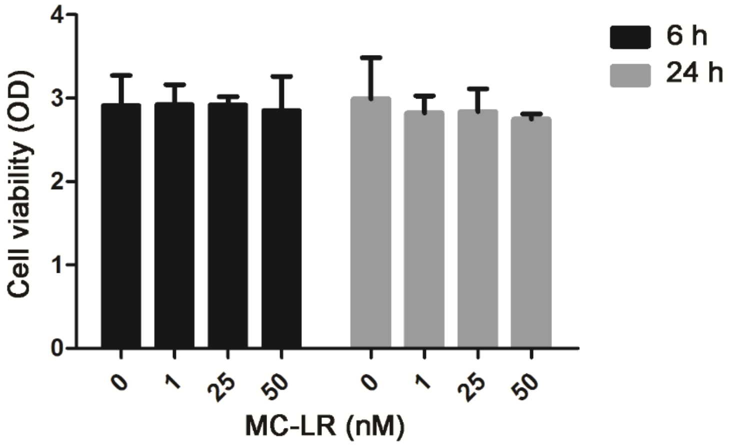

Figure S1. Toxic effects of MC-LR on GC-1 cells. Measurement of cell viability was carried out with CCK-8. The percentage of dead cells was increased in a dose-dependent manner.

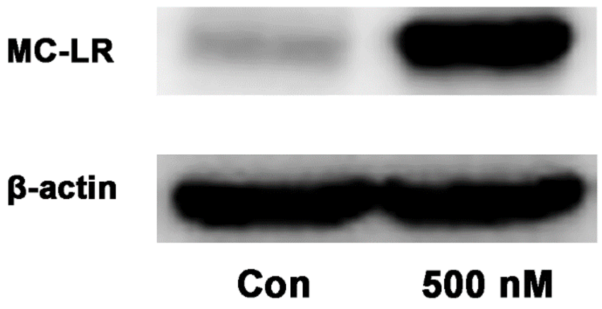

Figure S2. Intracellular MC-LR detected in GC-1 cells exposed to MC-LR for 24 hours. The protein levels of MC-LR in GC-1 cells treated with 0 (Con) and 500 nM MC-LR were detected by Western blot. β-actin was used as a reference.

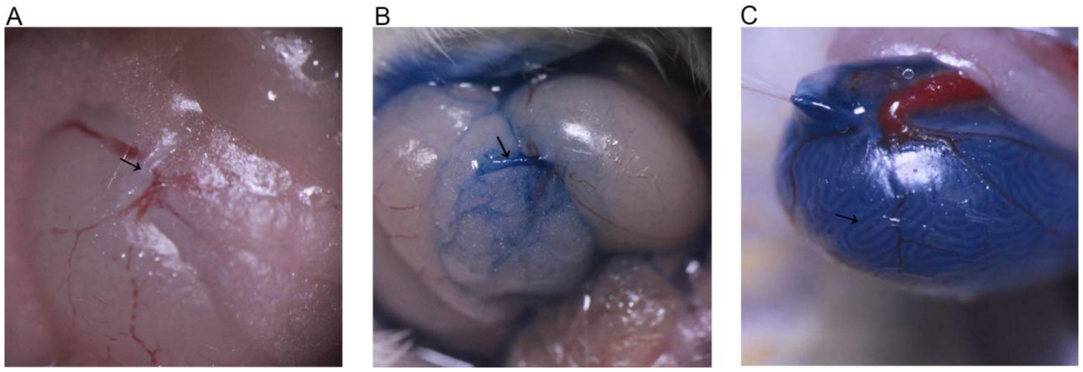

Figure S3. Efferent duct injection. In this technique, the micropipette is inserted into one of the large efferent ducts midway between the head of the epididymis and the testis. (**A**) The ducts are difficult to visualize (arrow); (**B**) The fatty tissue around the ducts has been removed. A small amount of blue dye solution injected between the ducts under the fibrous sheath may help to outline the ducts; (**C**) A

micropipette has been inserted into a large duct and threaded almost into the rete. Pressure in the pipette has been increased and the rete filled with dye solution. Tubules are also filled. And the tubules on the surface are almost totally blue indicating that the solution has entered and filled most if not all areas of seminiferous tubules. Flow of solution into the rete has generally stopped at this point. And the other efferent ducts as well as some portion of the epididymis head will also contain the injected solution.

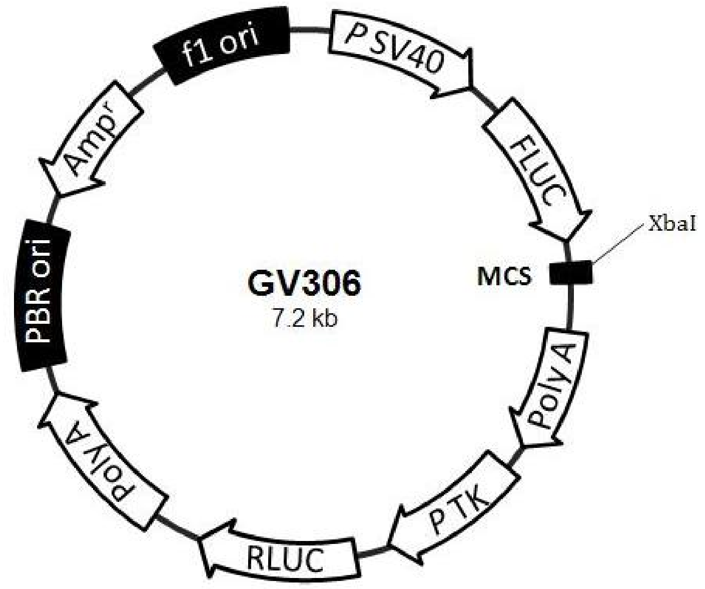

Figure S4. GV306 plasmid vector map. Gene fragment was cloned into vector by the two XbaI enzyme cleavage sites. The vector was purchased from Genechem, Inc. (Shanghai, China).

Table S1. Animals and treatment.

<table>
<tr><td>6-week-old mice</td><td rowspan="4">mice were acclimatized for 1 week before use</td><td colspan="2">intraperitoneally injection for 2 weeks</td><td colspan="3">collect the testicular tissues</td></tr>
<tr><td>group-1
group-2
group-3
group-4</td><td>7.5 μg/kg MC-LR
15 μg/kg MC-LR
30 μg/kg MC-LR
0 μg/kg MC-LR</td><td>MC-LR was dissolved in 400 μL physiological saline</td><td colspan="3">9-week-old mice</td></tr>
<tr><td>6-week-old mice</td><td>mice were given efferent duct injection on the first day of the seventh week.</td><td></td><td colspan="2">intraperitoneally injection for 2 weeks</td><td>collect the testicular tissues</td></tr>
<tr><td>group-5
group-6
group-7
group-8
group-9
group-10</td><td>miR-541-mimic
miR-541-inhibitor
mimic negative control
inhibitor negative control
miR-541-inhibitor
inhibitor negative control</td><td>mice were acclimatized for 1 week</td><td>0 μg/kg MC-LR

15 μg/kg MC-LR</td><td>MC-LR was dissolved in 400 μL physiological saline</td><td>10-week-old mice</td></tr>
</table>

Table S2. The sequence design of miR-541-inhibitor, inhibitor negtive control, miR-541-mimic and mimic negative control. They were purchased from Genechem, Inc. (Shanghai, China). miRNA-mimic, a kind of exogenous miRNA precursor, can play a similar role of endogenous miRNA after being cleavaged into mature miRNA in the host cells. It can increase the content of intracellular miRNA which down-regulates the protein level of its target gene. miRNA-inhibitor is a RNA segment which plays a role in combination of endogenous miRNA. After transfecting into the host cells, miRNA-inhibitor leads to the miRNA degradation and down-regulates the endogenous miRNA level. miRNA-Negative Control (miRNA-NC) is a meaningless nucleic acid sequence, which will not cause any changes of miRNA expression after entering into the host cells.

**miR-541-Inhibitor**
5′-AGTGTGACCAACATCAGAATCCCTT-3′
**inhibitor Negtive Control**
5′-CAGUACUUUUGUGUAGUACAA-3′
**miR-541-mimic**
ACCGGTTACATGAAGCTGCCCGTGGCGTCTTTTCCCCGTATGACACACTGCATGTGA CCAGTCTCTTGCTGCCCTGCAGCAAGGCTAGAAGCCTGTGTGAACTCTTGCCAAAA TCAGAGAAGGGATTCTGATGTTGGTCACACTCCAAGAGTTTTAAAATGAGTGGCGA ACACAGAATCCATACTCTGCTTATGGCCTAAGTCAATGGATCCTGACCCTTACTGG AGAGCCCTCCCTGGCGTGCTCGTGCGGGGCAGCCATACACACTGGCACAAGGGGA CCCTGCGGGATCTCCTGCTAGC
**Mimic Negative Control**
5′-TTCTCCGAACGTGTCACGT-3′

Note: The 5′ end underscored sequence is the AgeI restriction site, the 3′ end underscored sequence is the NheI restriction site, the sequence in red marker (miRNA precursor) is the coding region.

Table S3. Oligonucleotide Sequences used in this study.

Gene	Sequence 5′–3′
P15 (F)	AGCCAATCAGAAATAACTTCCTACGC
P15 (R)	GTACTGACTGCACCCACCCAAAT
GAPDH (F)	AACTTTGGCATTGTGGAAGG
GAPDH (R)	ACACATTGGGGTAGGAACA
Caspase 3 (F)	AGTGACCATGGAGAACAACA
Caspase 3 (R)	AGCTGCTCCTTTTGCTATGA
Cyt c (F)	AAATGGGTGGATGTTGAA
Cyt c (R)	GCCTCCTGGTGGTTAG
PP2A (F)	CAGAGCTCCAGAAAGC
PP2A (R)	TATTCAATGATGGCCAG
Bcl-2 (F)	CAAAGCCAAGCAGACG
Bcl-2 (R)	GCCCGACAATGGAGAA

Table S4. The wild type and mutant type of p15 3′UTR sequences containing the miR-541 binding site. The p15 3′UTR sequences containing the miR-541 binding site were constructed into a GV306 vector. The nucleic acids in frame are the binding site of miR-541. The nucleic acids in bold are the seed binding site mutated which was used as the Mut-p15 control.

Type	Sequence 5′–3′
p15 3′UTR	GAAAACUAUAAUUCUAAUUAACACUUAAAUAAUCAACAGU ACCUAAAAUGGAAUGUUUAGGGUUUUAUUAUUUUAAAUAA CUACAAUAGGGGGAAAAAAUCACACAGGUAGUAAUGUAAA CCUCUCACUUGAUUUAUAA**AAUCCCU**UAGCUUCUCCUUCAU UCCUUUUGGUCUAAAAUAUUUGAAUUUGCAAGUUGCAUUA UACUGGGUCAUGAAAAAUUAUCCCUUGAAAUAGAUAUGAA ACAUGUUACUUCAUUUCUGGU
Mut–p15 3′UTR	GAAAACUAUAAUUCUAAUUAACACUUAAAUAAUCAACAGU ACCUAAAAUGGAAUGUUUAGGGUUUUAUUAUUUUAAAUAA CUACAAUAGGGGGAAAAAAUCACACAGGUAGUAAUGUAAA CCUCUCACUUGAUUUAUAA**GGCUUUG**UAGCUUCUCCUUCA UUCCUUUUGGUCUAAAAUAUUUGAAUUUGCAAGUUGCAUU AUACUGGGUCAUGAAAAAUUAUCCCUUGAAAUAGAUAUGA AACAUGUUACUUCAUUUCUGGU
